# Progress on the Use of Commercial Digital Optical Disc Units for Low-Power Laser Micromachining in Biomedical Applications

**DOI:** 10.3390/mi9040187

**Published:** 2018-04-16

**Authors:** Aarón Cruz-Ramírez, Raúl Sánchez-Olvera, Diego Zamarrón-Hernández, Mathieu Hautefeuille, Lucia Cabriales, Edgar Jiménez-Díaz, Beatriz Díaz-Bello, Jehú López-Aparicio, Daniel Pérez-Calixto, Mariel Cano-Jorge, Genaro Vázquez-Victorio

**Affiliations:** 1 Facultad de Ciencias, Universidad Nacional Autónoma de México, Circuito Exterior S/N, Ciudad Universitaria, Ciudad de Mexico CP 04500, Mexico; aaron-cruz-27@ciencias.unam.mx (A.C.-R.); raulsan8@gmail.com (R.S.-O.); diego.zamarron@ciencias.unam.mx (D.Z.-H.); anlucato@ciencias.unam.mx (L.C.); edgarjd@ciencias.unam.mx (E.J.-D.); jehu@ciencias.unam.mx (J.L.-A.); daniel_perez@ciencias.unam.mx (D.P.-C.); marielcanoj@ciencias.unam.mx (M.C.-J.); genvazquez@ciencias.unam.mx (G.V.-V.); 2 Laboratorio Nacional de Soluciones Biomiméticas para Diagnóstico y Terapia LaNSBioDyT, Facultad de Ciencias, Universidad Nacional Autónoma de México, Circuito Exterior S/N, Ciudad Universitaria, Ciudad de Mexico CP 04500, Mexico; diazbello.b@gmail.com

**Keywords:** laser micromachining, scaffold, biomimetics, microadditive manufacturing, microsubtractive manufacturing

## Abstract

The development of organ-on-chip and biological scaffolds is currently requiring simpler methods for microstructure biocompatible materials in three dimensions, to fabricate structural and functional elements in biomaterials, or modify the physicochemical properties of desired substrates. Aiming at addressing this need, a low-power CD-DVD-Blu-ray laser pickup head was mounted on a programmable three-axis micro-displacement system in order to modify the surface of polymeric materials in a local fashion. Thanks to a specially-designed method using a strongly absorbing additive coating the materials of interest, it has been possible to establish and precisely control processes useful in microtechnology for biomedical applications. The system was upgraded with Blu-ray laser for additive manufacturing and ablation on a single platform. In this work, we present the application of these fabrication techniques to the development of biomimetic cellular culture platforms thanks to the simple integration of several features typically achieved with traditional, less cost-effective microtechnology methods in one step or through replica-molding. Our straightforward approach indeed enables great control of local laser microablation or polymerization for true on-demand biomimetic micropatterned designs in transparent polymers and hydrogels and is allowing integration of microfluidics, microelectronics, surface microstructuring, and transfer of superficial protein micropatterns on a variety of biocompatible materials.

## 1. Introduction

Biological cells are highly sensitive to geometrical cues and mechanical constraints from their microenvironment [1,2]. However, under classic culture conditions, the entirety of this spatial, chemical and mechanical information is lost as the cell microenvironment is a flat plastic or glass surface with a stiffness of a few gigapascal, several orders of magnitude greater than biological connective tissues and basal membrane [3]. Materials science and engineering, as well as microengineering techniques, provide tools to modify the properties of cell culture substrates at subcellular scales mimicking three-dimensional in vivo microenvironments [4], thus leading to better results in cellular biology [5]. Spatial confinement and cell confluence through microstructures such as microchannels and microwells has proven to be very important in mechanobiology [6] as it can promote cell-cell interactions, polarize the cells, control migration in the resulting biomimetic scaffolds [7]. However, an expensive infrastructure is usually required to develop the aforementioned biomimetic biological cell culture platforms, especially for on-demand designs and rapid prototyping. Recently, 3D printing has been growing to readily develop organ-on-chip platforms that offer variable results depending on the desired application [8], but machines typically lack micron-scale resolution or their cost is still very high.

Typically, large (centimeters) scale platform with high (micrometers) resolution are difficult to find at a low cost and in transparent materials as required in biology, thus limiting the development of new 3D platforms for cell culture and studies. Laser technology is a good alternative to resolve this particular limitation, as laser ablation and direct laser writing (DLW) are used in micromachining with a micron-range resolution in a great variety of substrates compatible with biology [9,10,11]. However, laser ablation typically requires expensive high-power, short-pulsed lasers, especially for transparent materials that are normally required in lab-on-chip and biological scaffold applications. DLW, although more affordable, is still too expensive for any laboratory. In this work, we present the recent progress made using a very low cost yet very robust CD-DVD-Blu-ray system for 2D and 3D micropatterning in biology-compatible materials. In our previous work, we had demonstrated that it was possible to use such a platform to microfabricate structures in poly-dimethylsiloxane (PDMS) [12]. By means of a thin light-absorbing carbon nanopowder layer coating a transparent polymer, it is indeed possible to convert the optical power of a strongly-focused low-power laser beam into a localized high temperature microplasma [13] and etch the surface of the material of interest. In this work, we present the recent progress made in structuring other transparent polymers that are widely available and useful in the construction of cell culture scaffolds and microfluidic platforms, either by direct laser etching using the near infrared (NIR) wavelength or direct laser writing using the Blu-ray (BR). We have also upgraded the previous system with a real computer numerical control (CNC) G-Code reader, to simplify and speed up the transfer and thus microfabricate the desired features on larger areas (of 25 mm × 25 mm and up to 7 cm × 7 cm with the help of manual stage). We report the successful patterning with micron-range features of poly-dimethylsiloxane (PDMS), poly-methyl methacrylate (PMMA), Loctite 3525 (Loctite), polyethylene terephthalate (PET) and poly-lactic acid (PLA), useful in the construction of biomimetic cell scaffolds. These latest results help us demonstrate the great potential of our low-cost laser alternative for on-demand fabrication of cell culture scaffolds in biomedical applications at a low-cost.

## 2. Manufacturing Principle Using a Low-Power Laser

### 2.1. Laser Ablation

The laser microablation system used in this work is based on a CD burner with a Lightscribe^®^ function as it offers greater power densities than other conventional CD burners. The optical pickup head (OPH) is extracted from the case and rails and placed as such on a 3D printed holder fixed to a motorized displacement stage. No modification is made to the pickup head as the original mounted lens is used to focus the near infrared (NIR) beam on the desired surface. Although both red and infrared lasers are available, only the latter was used here as it offered a better performance in etching more than the surface.

Although the laser power available with the laser diode of a CD-DVD pickup head is greatly limited, it was shown that high temperatures may be reached locally when it is shone onto absorbing nanocarbon [13]. This effect has been used in our group to laser-etch poly-dimethylsiloxane (PDMS) [12] and other transparent materials typically difficult to process and micromachine using lasers. By coating the desired material with a thin absorbing nanocarbon layer, the low-power laser can be focused precisely onto the surface to etch thanks to the ionization of the carbon that in turns ablates the surface of the substrate, using a method that had only been demonstrated with pulsed, high power lasers [14]. In this paper, three more materials tested with our method are presented, demonstrating its versatility.

In micromachining, control of the features resolution and geometrical characteristics is a critical parameter as it is the limiting factor of the applicability of resulting patterns and molds. As previously demonstrated in all the previous applications that were reported with our technique and setup, it was possible to obtain the desired features thanks to the instrumentation and control of the lasing conditions that are mentioned in the next section.

### 2.2. Direct Laser Writing

Similarly to the NIR laser OPH, a Blu-ray OPH (BR) was mounted on the same rails to use a strongly focused 405 nm laser beam for DLW applications on compatible photosensitive resins and materials. In order to validate the proof of concept and because DLW results are strongly dependent on the material to polymerize, we chose a cost-effective off-the-shelf photosensitive adhesive that is compatible with microfluidics platform construction and can be spin-coated to thin layers of a up to tens of micrometers: Loctite 3525 (Henkel, Düsseldorf, Germany). We conducted tests to verify that the control of laser power density enabled a good control of the dimensions and geometry of desired patterns in the micrometer range. After calibration, the control of the dose enabled the successful transfer of a 3D pyramidal Loctite micropattern on glass, in order to demonstrate the feasibility of 3D printing, at least up to a 20 µm height.

## 3. Instrumentation Considerations

### 3.1. CNC Platform

For the new experiments presented here, in order to optimize etching duration and working area, the setup and instrumentation used in our previous papers was modified. The two OPHs were still mounted on a three-axis motorized platform in a CNC fashion, with the XY stage holding the sample on which the laser may be focused thanks to the control of the Z stage. Displacements were controlled using bipolar microstepper motors (part number ZFS25B, Thorlabs Inc., Newton, NJ, USA) offering a one micron step in all directions and up to 25 mm travel in each direction. The setup is now driven by an electronic interface that is governed by the operator via a computing support where a g-code can be uploaded and followed in real-time on a display.

The control of the displacement platform used to etch an on-demand pattern on the desired substrates is achieved by communicating instructions to the motors thanks to Universal GCode Sender (Will Winder, released under the GPLv3 license) and an ATMega328 microcontroller (Microchip Technology Inc., Chandler, AZ, USA) loaded with GRBL CNC controller v1.1 (Simen Svale Skogsrud, Sungeun K. Jeon and Jens Geisler released under the GPLv3 license). In order to increase the writing speed without losing resolution and limit the possible mechanical load on the stepper motors, an A4988 chip (Allegro MicroSystems, Worcester, MA, USA) Double-diffused Metal-Oxide-Semiconductor (DMOS) microstepping driver designed to handle significant power levels with translator and overcurrent protection was used. This microstepping driver was employed as it can handle five different microstep resolutions from full to 1/16th full-step. In order to improve the step resolution, we use a 1/8th full-step which allowed us a theoretical resolution of 0.118 micron per step and a much smoother displacement and etching when compared to previous results. Pattern design was made using Inkscape and converted to G-Code using the J Tech Photonics Laser tool plugin for Inkscape. Finally, samples are held in place using a kinematic self-centering mount (part number KS1SC, Thorlabs Inc., Newton, NJ, USA). The footprint of the system is limited and adapted to any laboratory environment.

### 3.2. Lasing Conditions

An external circuit allows the selection of the laser diode, operation mode (continuous wave or pulsed) and pulse time. Laser power density is controlled with a built-in voltage controller, using a commercial diode current driver specially made for CD burners [15]. A calibration measurement of the laser power density (Lp) vs. electrical current (IL) has been achieved using an optical power-meter (part number PM1000, ThorLabs Inc., Newton, NJ, USA). It was then converted to a voltage to power density relationship as the machine offers a control of the electrical voltage to the operator. However, for reproducibility purposes and because OPHs may vary from batch to batch, the characterization presented here reports Lp as a lasing condition.

**Figure 1:**
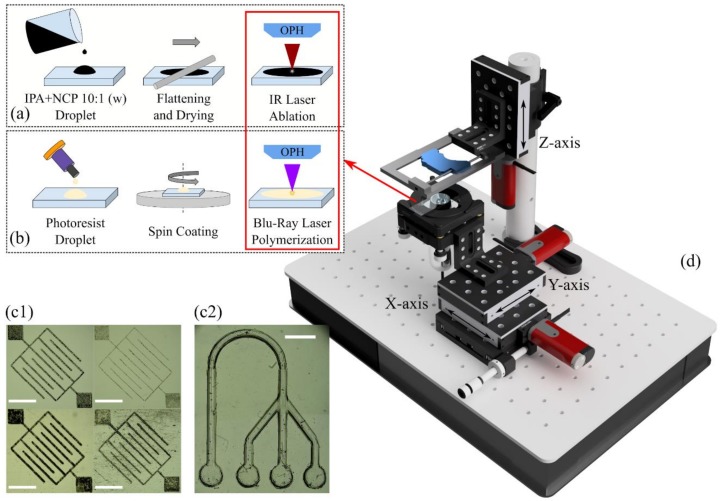
Diagrams of the laser ablation (**a**) and DLW (**b**) microfabrication methods to fabricate microstructures directly in transparent plastics such as PMMA, PDMS, PLA, and Loctite 3525 (**c1**) or by direct laser writing in Loctite 3525 (**c2**). Everything was achieved with the benchtop laser platform with two laser wavelengths (**d**). Electronics are not shown for clarity. Scale bars are 1 mm.

To ensure a correct focusing of the laser beam at the absorbing coating surface (Figure 1a) even during etching, the built-in integrated circuit photodiode (ICPD) embedded in the OPH was used as a focus-error-signal (FES) detector [16] connected to external amplifiers. After a straightforward calibration of the S-curve obtained from the signal, the resulting Z resolution was sufficient to implement an automated routine for each sample. Finally, a cost-effective portable USB microscope was located below the sample holder to visualize the process in real time.

### 3.3. Sample Preparation for Laser Ablation

As mentioned, absorbing nanocarbon materials are used in order to allow the subtractive manufacturing process to happen. The ionization process that etches the sample can be achieved with several nanocarbon types and it has been found that different allotropic forms may yield different resolutions and etching processes [17]. In this work, we report the characterization and progress made with carbon nanopowder (633100, Sigma-Aldrich, St. Louis, MO, USA). The material is mixed with a spatula in isopropyl alcohol (IPA) in 1:10 *w*/*w*, respectively, and deposited on the sample of interest with a doctor blading method. Samples are left to dry in the lab for a few minutes, leaving a homogeneously black, matte surface. Although a large variety of materials may be etched in this manner, the samples reported here are flat polymerized slabs of PDMS made from Sylgard 184 (Dow Corning Corp., Midland, MI, USA), flat PMMA sheets cut with a microscope slide dimensions, flat PLA sheets and UV-cured layers of Loctite 3525. All these samples were placed on a coverslip for manipulation, located on the sample holder ready for the autofocus routine. Once the process is terminated, the samples are washed with distilled water, sonicated, and dried with compressed nitrogen.

## 4. Characterization of the Manufacturing Processes

### 4.1. Laser Ablation Using NIR Laser

**Figure 2:**
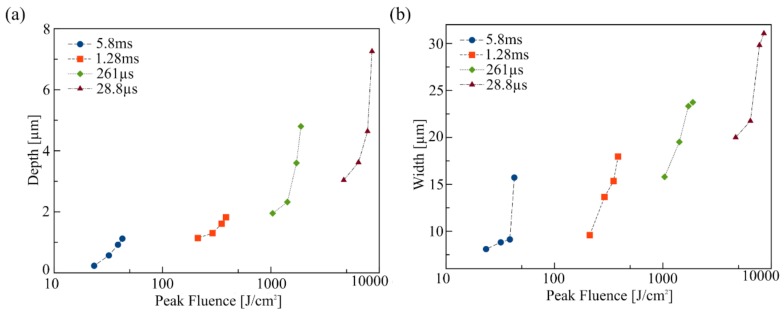
Laser-etching control in PMMA substrates: average depth (**a**) and width (**b**) as a function of laser power density and pulse time. Data points are averaged over the full patterns.

Lasing conditions like pulse or dwell time, power density, and number of passes are important to set when characterizing the ablation of a polymeric material using our method and setup. Figure 2 presents typical resolution characterization results obtained for PMMA using profilometry (AlphaStep^®^ D-600 Stylus Profiler, KLA-Tencor, Milpitas, CA, USA). An array of parallel lines was etched in an acrylic sheet at different laser intensities and pulse durations in order to monitor their influence on ablated patterns. As expected in laser ablation, it is clear that laser pulse time and power density are critical to control width (Figure 2a) and depth (Figure 2b) of the etched features for PMMA and this behavior was observed for all four tested materials here, although final features dimensions are different for each. This procedure is recommended to test our ablation method for each new material and repeatability and reproducibility may also be addressed in this manner.

As can be seen, etching depth is limited by the lasing characteristics for a given in-plane resolution and in general the depths that were yielded with our method were not acceptable for many applications such as microfluidics, biomimetic cell cultures, etc. As presented in Table 1, typical aspect ratios between depth and width (depth/width) measured by profilometry on straight lines ablated with different laser conditions resulted to be lower than 25% and an aspect ratio of 1 and above is desired for such applications. It is interesting to note that there is a clear difference from material to material (also visible in Figure 1c1) that are probably caused by a lower melting temperature (PMMA) or in-plane deformations of the material coated with nanocarbon under NIR light causing a deeper penetration of the etching energy (in case of PDMS and PLA). For more rigid materials, such as crosslinked Loctite or PET, etching was only superficial. PET sheets were also only deformed at greatest laser power densities, as profilometry showed large protrusions inside the etched micropatterns.

**Figure 3:**
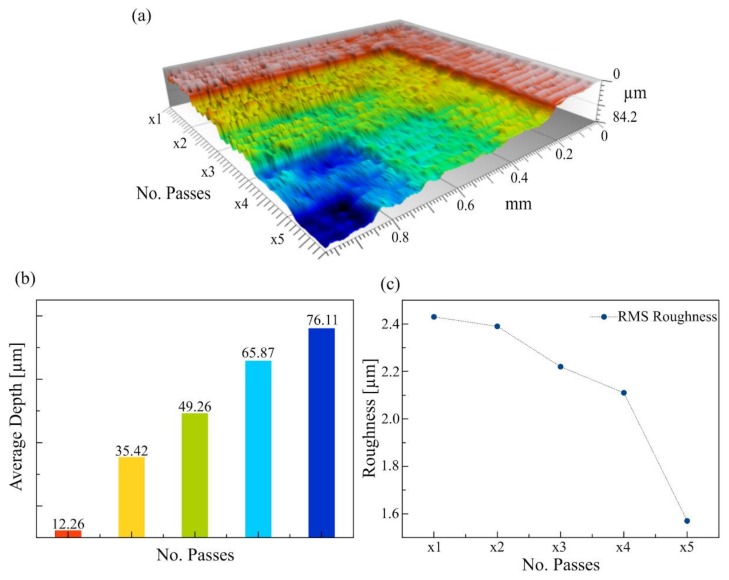
Example of a 3D profile obtained by ablation with multiple laser passes: (**a**) 3D profile of the successive etched layers; (**b**) the average depth of a square region etched one to five times (1X to 5X); and (**c**) the Root Mean Square roughness of the etched area as a function of the number of passes.

As can be seen, the depth of ablated geometries was very limited. However, this was remedied by repeating the coating-etching-washing steps of our procedure several times: it was possible to etch the same region with the desired pattern several times and thus control the depth by the number of passes. Figure 3 presents the results obtained for PMMA with the maximum laser power density and greatest pulse time. In this experiment, in-plane resolution of individual etched pixels was not important. The linear behavior of resulting depth vs. the number of passes is visible up to five passes and dimensions compatible with the construction of cellular scaffolds were reached. Moreover, when the final application of the etched patterns requires a smoother surface, it was found that multiplying the number of laser passes with or without renewing the coating reduces the roughness as well.

**Figure 4:**
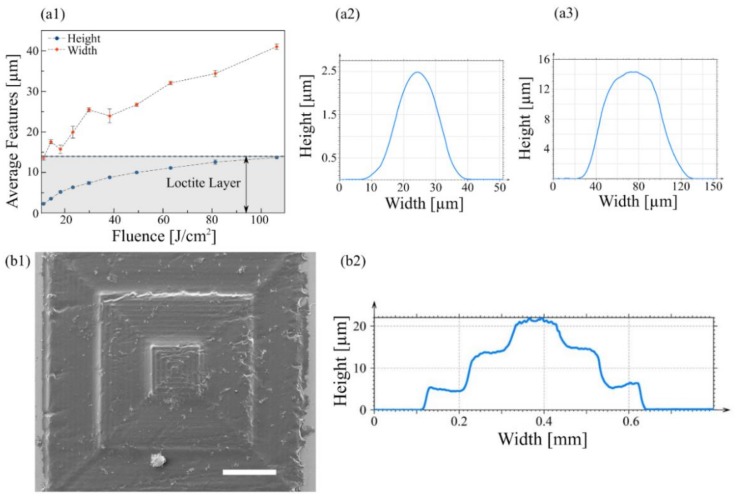
(**a1**) Dimensions of crosslinked features on Loctite 3525, and profiles of the (**a2**) smallest and (**a3**) tallest features fabricated with 4 W/cm^2^ and 106 W/cm^2^ respectively. (**b1**) SEM micrograph of a 3D pyramid fabricated on a single layer of Loctite 3525 by crosslinking the material in a Z-stepper fashion with a laser power density of 37.05 W/cm^2^; and (**b2**) the profile measured at the middle of the pyramid.

Thanks to the characterization of the process for each material, it was then possible to etch different features in each material for comparison. Figure 4 presents micrographs of a similar pattern of interdigitated parallel lines in the four materials of interest tested here for a laser power density of 576 mW/cm^2^ and a pulse time of 5.8 ms (one laser pass). As the most flexible material, PDMS yields finer features but much less deep than other, more rigid materials.

### 4.2. Additive Manufacturing Using Blu-Ray Laser

In addition to the NIR laser diode that we have previously reported, a commercial BR OPH was mounted on the CNC platform for additive manufacturing using a UV-blue photosensitive material. As reported in the literature [18], the Loctite 3525 adhesive resin is an excellent cost-effective candidate for such an application as it is widely available, compatible with microfluidics applications and it develops readily in off-the-shelf acetone instead of very aggressive solvents. A homogeneous layer of Loctite 3525 was spin-coated on a glass slide and polymerized locally using the blue laser beam.

To test the dependency of the dimensions of crosslinked features with laser power density, the lens was approximated at the focal distance using the same auto-focus sequence as previously described for NIR ablation and parallel lines were transferred successfully after the non-crosslinked resin was removed by acetone. Figure 4 shows a clear increase of width and height of the transferred patterns measured by profilometry up to the layer thickness of the adhesive (14 µm). It was interesting to remark that the profile of such patterns appeared to be of Gaussian shape and that this was even conserved at the greatest power density.

We also used the setup for 3D fabrication of more complex patterns: instead of piling up and micropatterning several 2.5D layers in a consecutive fashion, a pyramid geometry was fabricated in a spin-coated Loctite layer, crosslinked directly in one run, similar to a 3D printer, using our planar CNC for each plane and the Z-axis to polymerize on top. Figure 4 shows that by using the laser conditions necessary to transfer 5 µm-high patterns and a correct 3D construct slicing/Z-axis displacement, it was possible to imprint in three dimensions. It was interesting to remark that the total height of the pyramid overtook the original layer thickness. This was probably caused by a reshaping or volume increase of the viscous non-crosslinked resist as the lowest polymerized slices were stiffening due to light absorption. More work will be done in the future to test the feasibility to release 3D structures, like beams and cantilevers.

## 5. Examples of Successful Applications

PMMA offered favorable results with our laser technique to fabricate micromolds for scaffold applications. In this section we present a brief review of what has been achieved with this material.

### 5.1. Microelectrodes

In electronic devices, like sensors and biosensors, microelectrodes are useful and necessary to obtain a signal from non-metallic elements, like cells or biological analytes. In biosensors, to avoid any type of bioactivity, microelectrodes are made of inert materials, like silver, gold, platinum, stainless steel, or carbon paste. Microelectrodes are used in applications like voltammetry, amperometry and impedance.

**Figure 5:**
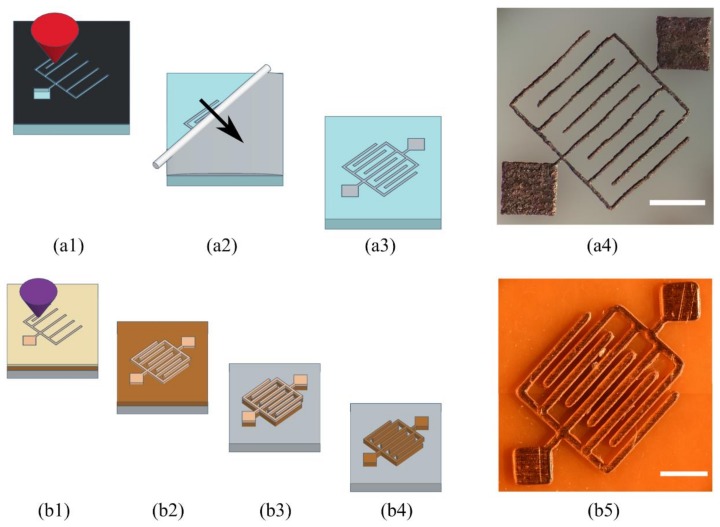
Microelectrodes fabrication processes: local laser ablation of PMMA etches interdigitated microchannels that are filled with carbon paste using doctor blading (**a1**–**a4**) and DLW is used on a thin Loctite layer as a protective coating to chemically etch a PCB copper board (**b1**–**b5**) Scale bars are 500 µm and 1 mm for (**a4**) and (**b5**) respectively.

We used our laser ablation technique to produce microchannels on PMMA substrates with controlled geometry. Those microchannels were filled with a conductive material that was converted into microelectrodes of desired shapes and geometries after drying. Geometrical and electrical characterization of these structures were already reported [19]. To validate our process, different geometries, sizes, and materials were used as electrodes, and then tested for amperometry and impedance measurements. By using our technique, it was possible to produce electrodes made of carbon paste and silver to design a glucose sensor. Furthermore, it was possible to fabricate a microelectrode array, as shown in Figure 5.

### 5.2. Microfluidic PMMA Molds

**Figure 6:**
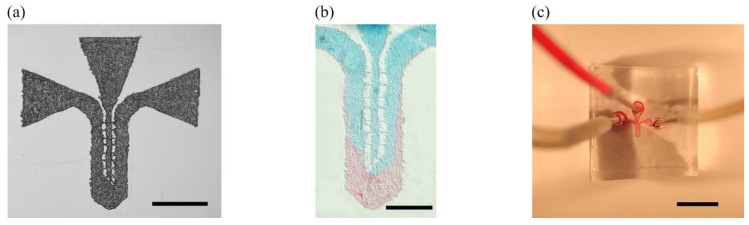
(**a**) Laser micropatterned PMMA mold for two-input two-output microfluidic channel design. (**b**) Example of colorant diffusion microfluidics inside PDMS microchannel for bonding validation. (**c**) Microfluidic device (Scale bars: (**a**) 1 mm, (**b**) 500 µm, (**c**) 5 mm).

Soft lithography with PDMS has been widely used in the development of microfluidic chips. Among their many applications, microfluidic chips for cell culture stand out as innovative tools allowing cell microenvironment purpose studies [20]. Our low-cost laser ablation technique can be used to fabricate specifically-designed molds on PMMA substrates, which may be then replicated and sealed with PDMS in order to fabricate microfluidic devices. Based on a published work where a biomimetic liver lobule was constructed from an SU8 mold obtained with a conventional photolithographic technique [21], we reproduced a mold consisting of two channels interconnected to each other by slits, by directly patterning the desired design onto a PMMA substrate (Figure 6). A double PDMS replica of our created mold was required in order to achieve a channel-like microfluidic structured PDMS surface.

### 5.3. PDMS Micropatterns for Selective Biological Cell Regional Growth

**Figure 7:**
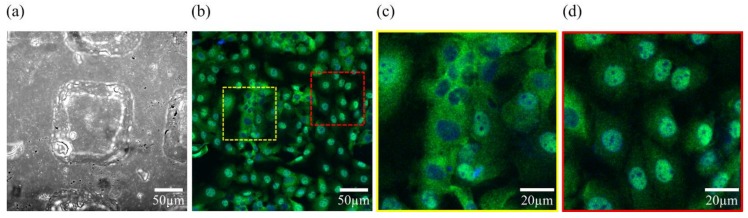
(**a**) Bright field photograph of a squared microwell. (**b**) Regionalized hepatic C9 cells cultured onto PDMS replica, (yellow discontinued square corresponds to (**c**) and the red one corresponds to (**d**)). (**c**) In response to spatial confinement, cells upon/atop the microwell present YAP/TAZ (green) in the cytoplasm as shown by the non-colocalization with the nuclei stained with DAPI (blue). (**d**) On the contrary, cells outside the microwell, have YAP/TAZ in the nuclei, as shown by the colocalization with DAPI (full experiment unpublished at the time).

The biocompatibility and accessibility of PDMS is very promising for this type of biomedical research and developing polymer—based tissue engineering platforms and PMMA molds offered better results for PDMS structuring. For selective regional growth it is quite relevant not only the control of features such as size and geometry, which are controlled by our method, but also the capability to improve cell adhesion with the carbon nanodomains within the etched channels as a result of laser ablation [17]. To validate our technique, hepatic C9 cells were seeded on the microstructured PDMS (Figure 7). By manipulating micropattern shapes, cells seem to adapt their architecture to the geometry of the microenvironment where they are found. In addition to the cellular morphological appearance, immunostaining was performed in order to visualize YAP (Yes-associated protein)/TAZ (Tafazzin) proteins localization inside the cell. nuclear translocation of YAP/TAZ is present in undifferentiated and proliferative cells. It is known that the subcellular localization of these proteins could change in response to external signals such as stiffness and confinement [22]. Cells inside the microwells present YAP/TAZ proteins in the cytoplasm as is shown by the non-colocalization with the nucleus marker DAPI (Diamidino-2-phenylindole) in Figure 7. YAP/TAZ protein sequestered in cytoplasm is a consequence of cell compaction and likely a less proliferative cells.

In future perspectives for biomimetic cell culture platforms fabrication, it is mandatory to understand how the geometry of the microenvironment impacts the cellular physiology to develop more accurate in vitro models for investigation, such as drug testing.

### 5.4. Microcontact Printing Stamps

**Figure 8:**
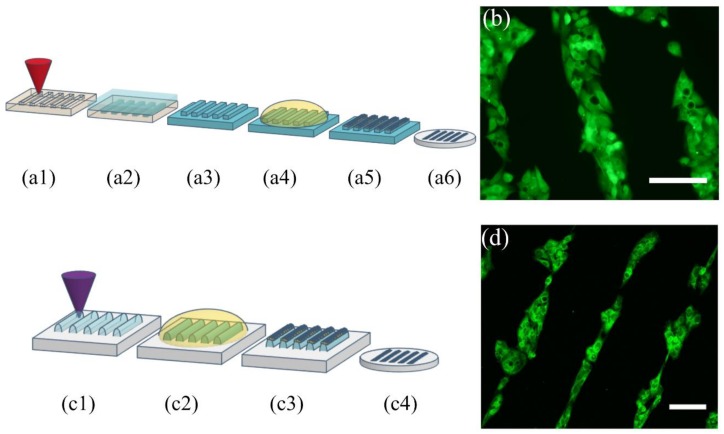
Diagram of PMMA laser etching for the fabrication of µCP master molds: (**a1**) Laser etching of PMMA to obtain a mastermold, (**a2**) PDMS is poured onto the PMMA structured master, (**a3**) the stamp is peeled off the master, (**a4**) the stamp is incubated with an ink solution, (**a5**) the excess of solution is removed, (**a6**) the inked stamp is placed onto a glass coverslip and the protein pattern is suitable for cell culture. (**b**) HepG2 cells grow selectively onto the collagen I protein pattern (green is calcein (live cells), scale bar: 100 µm. Diagram of Loctite DLW for the fabrication of µCP stamps in one single step: (**c1**) Selective laser crosslinking of Loctite to obtain the desired stamp, (**c2**) the stamp is incubated with an ink solution, (**c3**) the excess of the solution is removed, (**c4**) the inked stamp is placed onto a glass coverslip and the protein pattern is suitable for cell culture. (**d**) HepG2 cells onto the transferred protein pattern (green is phalloidin as actin cytoskeleton marker), scale bar: 100 µm.

Microcontact printing (µCP) is a soft lithography technique that is used to transfer patterns of an “ink” using a PDMS stamp [23]. Stamps are usually fabricated by replicating the microstructures of a homogeneous SU-8 master mold for superficial transfer and patterning of protein, DNA, cells, or other molecules. Although it is known that the quality of the surface of the stamp is critical for an adequate transfer of micropatterns, we have successfully used the relatively rough PMMA molds to fabricate PDMS stamps (Figure 8a), as well as Loctite stamps made by one single DLW step, without further treatment (Figure 8c) for protein transfer on PDMS substrate for collagen-selective HepG2 cell culture. It was interesting to note that the hydrophilic nature of the surface of crosslinked Loctite did not require any treatment or modification before nor expanding of the protein droplet during inking. Additionally, Loctite 3525 is much more rigid than PDMS and its DLW structures were Gaussian-shaped, hence, it offered much less deformation during microcontact printing while maintaining a shorter contact area for the formation of single-cell lines. This is of particular importance as the possibility of confining cells within a controlled geometry is very useful in studies of cell adhesion, cell-cell interactions, tissue engineering and in the development of cell-based biosensors. In this work, although PDMS substrates could be etched directly to obtain the desired relief patterns before inking, the resulting depth has not proven to be efficient in transferring the pattern to another PDMS substrate. It was indeed much more effective when the stamps were obtained from a PMMA micromold with a 50 µm depth or with Loctite. The validation example that was successfully made in our group was that of collagen type I inking of PDMS stamps for regionalized cell growth and alignment [24]. Following the process previously described, HepG2 cells were successfully patterned onto soft and stiff PDMS surfaces as well as glass coverslips (Figure 8). It is important to note that all the µCP patterns were not rinsed away during the cell medium incorporation or washing. For future applications, PDMS stamps with more complex geometries can be achieved with our method.

### 5.5. Hydrogel Microstructures

**Figure 9:**
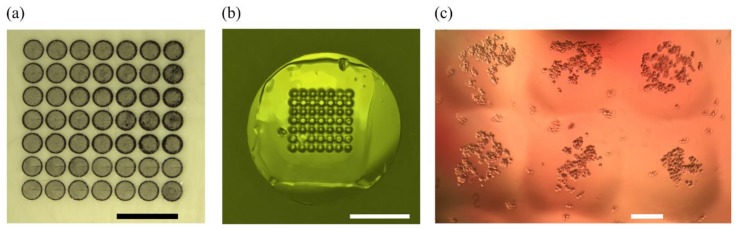
Polyacrylamide hydrogels with microposts structures: PMMA laser-etched mold with microwells (**a**), vertical microposts are transferred to polyacrylamide (**b**) and A549 cells were seeded on 300 micron wide hydrogel pillars for confinement studies (**c**). Scale bars are 3 mm for (**a**), 1 cm for (**b**), and 100 µm for (**c**).

Hydrogels are also very important materials for scaffold fabrication. For instance, polyacrylamide is offering interesting mechanical properties for cell culture, it is easy to manipulate and microstructure to use in the construction of biomimetic scaffolds for stiffness assays [25]. With our laser etching technique, it was also possible to use PMMA micropatterned molds to fabricate 2.5D microstructures made of polyacrylamide hydrogels for cell culture platforms of desired geometries using a simple soft lithography technique of replica-molding (Figure 9). Indeed, the molds were used to pattern the gels during photo-crosslinking by sandwiching the hydrogel between laser-etched PMMA mold (Figure 9a) and a glass coverslip to obtain a microstructured hydrogel surface, ideal for cell culture patterning and confinement (Figure 9c). Preliminary cell culture results are very promising and will be presented elsewhere in a near future.

## 6. Conclusions

We have reported interesting results of the use of a low-cost laser platform consisting of a BR OPH for selective and localized polymerization of photosensitive materials and a CD burner to etch transparent materials for the fabrication of high-resolution molds in different materials. By setting the correct lasing conditions, our method enables the control of transfer of micron-range feature useful for biomimetic scaffold construction. The microstructuring of such materials is opening this field of research and its applications to low-budget laboratories, where the fabrication of micromolds using photolithography techniques is limited. Rapid prototyping of PMMA, PDMS, Loctite 3525, and PLA at a very low-cost and in short time using the commercial laser makes it a good alternative to other techniques for direct laser etching at the micrometer scale of the desired substrates or for a hard mold used in soft lithography.

## Figures and Tables

**Table 1:** Best aspect ratios for different plastic substrates. Superscripts are used to show the corresponding laser power density of each case.

Materials	Laser Pulse Duration			
	5.8 ms	1.28 ms	261 µs	28.8 µs
PMMA	23% ^1^	20% ^1^	12% ^4^	10% ^2^
PDMS	11% ^1^	11% ^2^	12% ^2^	8% ^3^
PLA	11% ^1^	16% ^2^	18% ^2^	15% ^2^
Loctite 3525	3% ^2^	4% ^2^	3% ^3^	8% ^3^
PET	4% ^2^	1% ^2,3^	N/A	N/A

^1^ @739 kW/cm^2^, ^2^@672 kW/cm^2^, ^3^ @554 kW/cm^2^, ^4^ @405 kW/cm^2^.
